# Metabolic engineering of soybean for improving grain quality for animal consumption

**DOI:** 10.3389/fpls.2026.1738805

**Published:** 2026-02-18

**Authors:** João Matheus Kafer, João Vitor da Silva, Suéllen Rosa de Almeida Polizeli, Rodrigo Thibes Hoshino, Juliana da Rosa, Elibio Leopoldo Rech Filho, Alexandre Lima Nepomuceno, Liliane Marcia Mertz-Henning

**Affiliations:** 1 Department of General Biology, Londrina State University, Londrina, PR, Brazil; 2 Embrapa Soja, Londrina, PR, Brazil; 3 Embrapa Genetic Resources and Biotechnology, Brasilia, Distrito Federal, Brazil

**Keywords:** antinutritional factors, CRISPR/Cas9, protein enhancement, RNA interference, soybean biotechnology

## Abstract

Soybean is one of the main sources of vegetable protein used in animal feed, but its nutritional value is limited by the presence of antinutritional factors, such as protease inhibitors (Kunitz and Bowman–Birk), lectins, phytic acid, raffinose family oligosaccharides (RFOs), and saponins, which reduce the digestibility and absorption of nutrients. In recent decades, advances in metabolic engineering and functional genomics have allowed the targeting of biochemical pathways to increase the content and quality of proteins while simultaneously reducing these undesirable compounds. This work reviews the main progress achieved through transgenesis, induced mutagenesis, and precision gene editing, highlighting the role of tools such as RNAi, CRISPR/Cas9, and AlphaFold2-guided gene editing in modifying genes involved in carbon and nitrogen metabolism and storage proteins. Recent studies demonstrate that the silencing of negative regulatory genes, such as *CIF1* and *AIP2*, can elevate the protein content of seeds, while the editing of sugar transporters *SWEET10a* and *SWEET10b* allows the modulation of the oil-protein balance. Simultaneously, the inactivation of genes related to antinutritional factors has significantly reduced the expression of compounds such as phytate and protease inhibitors. The integration of new approaches, such as promoter engineering and Prime Editing, promises to further enhance the precision of genetic modifications, minimizing pleiotropic effects. Taken together, these strategies consolidate metabolic engineering as a promising tool for the development of soybean cultivars with higher protein content and quality, and with lower content of antinutritional factors, optimizing their use in animal feed

## 1 Introduction

Soybean [*Glycine max* (L.) Merr.] is the primary cost-effective source of protein used worldwide. Soybean processing generates a range of by-products, including flour, oil, and lecithin, among others (Alarape et al., 2024; Oviedo-Rondón et al., 2024). Soybean meal, a by-product obtained from oil extraction, is a high-quality protein source containing approximately 48% crude protein. It is characterized by high digestibility and a favorable amino acid profile, providing essential amino acids such as lysine, tryptophan, threonine, isoleucine, and valine, which are typically limited in cereal-based diets (Lagos and Stein, 2017; Sotak-Peper et al., 2017). In addition, soybean meal provides metabolizable energy due to its residual lipid content and supports digestive health through the presence of insoluble fiber. These properties make soybean meal a key ingredient in feed formulations for poultry, swine, and ruminants. However, the nutritional quality of soybean grains can be influenced by theirs protein composition and the occurrence of antinutritional factor that may impair nutrient digestibility and absorption (Huang et al., 2023; Toomer et al., 2023; Xiao et al., 2025).

Antinutritional factor (ANFs) are detrimental in animal feeding, particularly for monogastric species such as poultry and swine (Nahashon et al., 2013). In soybean, the main ANFs of nutritional relevance include lectins, saponins, enzyme inhibitors, phytates and raffinose family oligosaccharides, all of which substantially reduce the nutritional value of soybean meal (Di et al., 2024). These compounds interfere with the activity of digestive enzymes, cause damage to the intestinal epithelium, decrease nutrient digestibility, trigger inflammatory responses, and consequently impair feed conversion efficiency in animals (Liener, 1994; Samtiya et al., 2020).

In the industry, soybean meal and its derivatives intended for animal consumption undergo heat treatment to eliminate antinutritional factors (Thakur et al., 2022). However, excessive processing can affect protein solubility, thereby reducing amino acid bioavailability and ultimately compromising the quality of soybean-derived proteins (Parsons et al., 1992; van den Berg et al., 2022). Thus, heat treatment increases production costs and can negatively impact the nutritional quality of soybean meal.

Protein content and amino acid composition are critical factors in formulating animal feed derived from soybean grains. Protein concentration can vary depending on genotype, soil profile, and nutrient availability (Guo et al., 2022). Although soybean protein is rich in some essential amino acids, such as lysine, it is not considered a complete protein (Patil et al., 2017; Singer et al., 2019, 2023). Soybean meal is particularly deficient in sulfur-containing amino acids, such as methionine and cysteine, thus requiring supplementation when used as the sole protein source (Panthee et al., 2006). Therefore, the development of soybean cultivars with low levels of antinutritional factors and high protein content, combined with a balanced composition of essential amino acids, is essential to meet industrial demands and to minimize adverse effects on animal health.

New Breeding Technologies (NBTs) have been employed to enhance soybean traits for animal nutrition. Over time, approaches such as mutagenesis, marker-assisted selection, genomic selection, transgenesis, genetic modifications using CRISPR-Cas and RNA interference have significantly contributed to the development of cultivars with optimized protein profiles and reduced concentrations of antinutritional compounds. Within this framework, the present review aims to summarize the major advances achieved in improving soybean protein quality and reducing antinutritional factors through the application of NBT-based strategies.

## 2 Soybean importance and composition

The popularity of soybeans is due to their high nutritional value, making them a fundamental food source for both human and animal consumption (Sudarić, 2020; Deng and Kim, 2024). Their composition is well balanced, containing significant amounts of proteins, fats, and carbohydrates. With approximately 35–40% protein in their dry weight, soybeans are an excellent source of protein, providing all essential amino acids (Sudarić, 2020; Modgil et al., 2021). This makes them comparable to animal-derived proteins and highly suitable for vegetarian and vegan diets. In addition, soy protein is widely used in the processed food industry, such as in tofu, soy milk, and protein isolates (Sudarić, 2020; Modgil et al., 2021; Deng and Kim, 2024).

Regarding fat content, soybeans contain about 18–20% lipids, which are predominantly unsaturated fats, including omega-3 and omega-6 fatty acids that play an important role in cardiovascular health (Hymowitz, 1970). Soybeans also contain lecithin, a phospholipid extensively used in the food and cosmetics industries (List, 2015). Carbohydrates account for roughly 30% of soybean composition, with a notable proportion of dietary fiber, which promotes digestive and intestinal health (Medic et al., 2014). Polysaccharides such as cellulose, and oligosaccharides such as raffinose and stachyose, are also present in significant amounts, although they can cause gastrointestinal discomfort in some cases (Dierking and Bilyeu, 2008; Zeng et al., 2021; Elango et al., 2022).

In addition to these macronutrients, soybeans are rich in bioactive compounds such as isoflavones (genistein, daidzein, and glycitein), which act as antioxidants and may contribute to bone and cardiovascular health, as well as provide benefits in hormonal regulation (Yamagata, 2019; Kanadys et al., 2021; Fatima et al., 2025). Another class of compounds found in soybeans are phytosterols, which help reduce LDL cholesterol (low-density lipoproteins) in the blood, benefiting heart health (Pelletier et al., 1995; Matvienko et al., 2002; Ilha et al., 2020).

Soybeans are also an excellent source of vitamins and minerals, being rich in B-complex vitamins, vitamin E, and minerals such as iron, magnesium, and phosphorus. These nutrients make soybeans a highly nutritious and functional food, also providing calcium, potassium, and phosphorus, which are essential for bone strengthening, muscle function, and fluid balance in the body (Kumar and Pandey, 2020).Additional compounds such as phytic acid, lectins, and saponins are considered antinutritional factors, as they can reduce the bioavailability of minerals, interfere with protein digestibility, and, in some cases, affect intestinal absorption (Viteri, 1997; Lott et al., 2000; Shi et al., 2004). However, these compounds have also been reported to possess certain biological activities, such as antioxidant and immunomodulatory effects, which are currently being investigated for potential health benefits when present in controlled amounts lipoxygenases (Liener, 1994; Samtiya et al., 2020; Salim et al., 2023; Di et al., 2024).

Therefore, soybeans are an extremely nutritious food with a diverse composition that offers numerous health benefits. Their content of protein, healthy fats, fiber, vitamins, and bioactive compounds makes them an excellent option for both human diets and animal nutrition (Samtiya et al., 2020; Toomer et al., 2023; Deng and Kim, 2024; Fatima et al., 2025).

### 2.1 Soybean processing for animal feed

Soybean is widely used in animal nutrition due to its high protein and essential amino acid content, being one of the main raw materials in feed formulation (Banaszkiewicz, 2011; Samtiya et al., 2020; Lambo et al., 2023; Salim et al., 2023; Xue et al., 2023). After harvesting, the grains go through cleaning, grinding, and oil extraction steps, which can be performed by mechanical pressing or by solvents, such as hexane. The byproduct resulting from this process is soybean meal, a fundamental ingredient in the feeding of swine, poultry, and ruminants, due to its high digestibility and biological value (Banaszkiewicz, 2011; Samtiya et al., 2020; Lambo et al., 2023; Salim et al., 2023; Xue et al., 2023).

For soybean meal to be suitable for animal consumption, it is essential that it undergoes controlled heat treatment, usually through toasting. This process aims to inactivate heat-labile compounds naturally present in the raw grains, which can interfere with nutrient digestion and absorption (González-Vega et al., 2011; Sotak-Peper et al., 2017). The toasting temperature and time must be carefully adjusted: insufficient heating (underprocessing) can leave residues of undesirable compounds that reduce protein digestibility, while overheating (overprocessing) can denature proteins, decrease the availability of essential amino acids, and compromise the nutritional value of the meal (Lagos and Stein, 2017; Oliveira et al., 2020).

Adequate thermal processing significantly improves the biological value of the protein, feed efficiency, and digestive safety of the animals. On the other hand, the use of raw or poorly processed soybean can lead to reduced weight gain, low feed conversion, diarrhea, and gastrointestinal problems, especially in young or monogastric animals (González-Vega et al., 2011; Sotak-Peper et al., 2017; Oliveira et al., 2020; Ma et al., 2022).

After heat treatment, soybean meal is mixed with other ingredients, such as corn, wheat bran, minerals, and additives, for the formulation of balanced feeds. Frequently, this mixture is subjected to pelletizing, a process that uses heat and pressure to compact the ingredients into pellets, facilitating transport, reducing segregation, and increasing the digestibility of starch and protein (Huang et al., 2023; Toomer et al., 2023; Xiao et al., 2025).

Although thermal processing increases the overall production cost of soybean-based feeds, the investment is economically justifiable due to the improvements in nutritional quality and animal performance it provides. According to recent industrial estimates (How Much Does a Soybean Oil Processing Plant Cost?, 2024), the operational cost for processing soybeans—including cleaning, dehulling, oil extraction, and heat treatment—ranges from US$ 30 to US$ 50 per metric ton, depending on plant scale, energy efficiency, and solvent recovery technology. These values align with the additional processing costs typically associated with toasting and conditioning required to inactivate heat-labile antinutritional factors in soybean meal. Despite these added expenses, proper thermal treatment significantly enhances protein digestibility, amino acid availability, and feed conversion efficiency, resulting in better zootechnical performance and overall economic return in animal production systems.

## 3 Antinutritional factors

**Figure 1:**
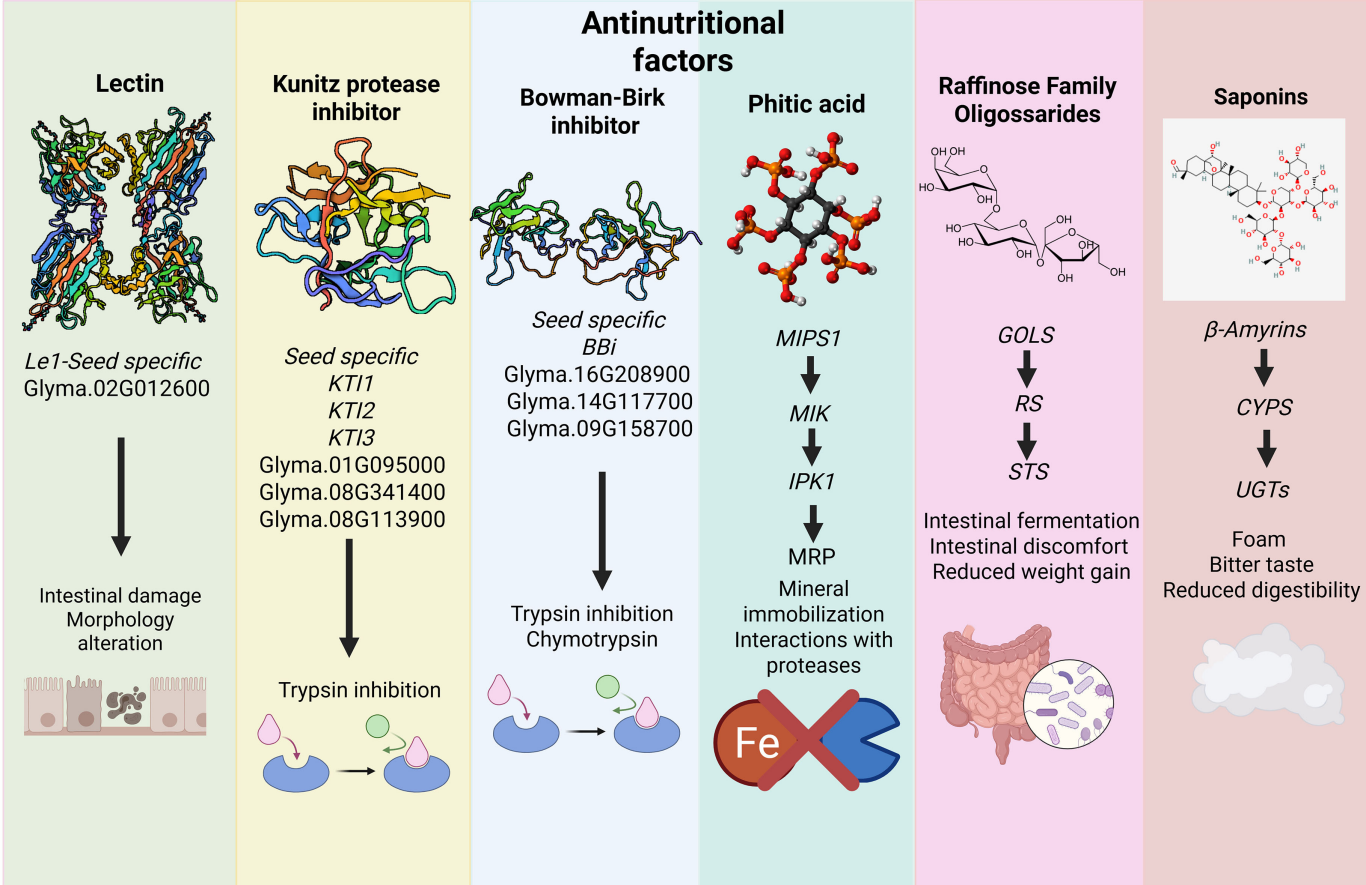
Overview of major antinutritional compounds in soybean and their molecular pathways.

Despite the benefits of soybean consumption, it contains several components that can cause harm when consumed. These are called antinutritional factors (ANFs), which are defined as “compounds that occur naturally or synthetically that interfere with the absorption, digestion, or utilization of nutrients by a body” (Liener, 1994; Woyengo et al., 2017). The main antinutritional factors present in soybean are trypsin inhibitors and lectins (Vasconcelos et al., 2006; John et al., 2021). Antinutritional factors can be classified as thermostable and heat-labile. In soybeans, the thermostable ANFs are saponins, tannins, oligosaccharides, allergens, and phytate, while the heat-labile ANFs are trypsin inhibitors, lectins, ureases, and lipoxygenases (Liener, 1994; Samtiya et al., 2020; Salim et al., 2023; Di et al., 2024). The antinutritional factors discussed in this review are summarized in **Figure 1**.

Lectins (hemagglutinin or agglutinin) are one of the main antinutritional factors in soybeans, containing 10 to 20 g.kg^-1^ of lectin, considering that a lectin concentration greater than 7 g.kg^-1^ is considered harmful to digestibility and unsuitable for direct consumption (George et al., 2008). In soybean, the seed lectin is controlled by the single *Le1* gene (Glyma.02G012600). In *le1* genotypes, a 3.5-kb insertion element disrupts the gene’s reading frame, preventing lectin from accumulating in the seeds (Goldberg et al., 1983; Vodkin et al., 1983; Vodkin and Raikhel, 1986; Okamuro and Goldberg, 1992; Shamimuzzaman and Vodkin, 2018). Lectin is a tetrameric glycoprotein of four identical subunits, each 30 kDa, totaling 120 kDa, which can reversibly bind to carbohydrates (Olsen et al., 1997; Sharon and Lis, 2001; Halder et al., 2016).Binding to carbohydrates occurs through Ala-86, Asp-88, Ala-105, Phe-128, Asn-130, Leu-214, Asp-215, and Ile-216, which can interact with intestinal cells, causing damage and changing their conformation. Furthermore, when consumed, the protein is not digested by proteases in the gastrointestinal tract and undergoes endocytosis in the intestine (Ma and Wang, 2010; Pan et al., 2013). When lectin enters the cell, it induces hyperplasia and hypertrophy of the small intestine. Several articles have demonstrated decreased performance in monogastric animals when fed raw soybean seeds. In pigs, soybean agglutinin (SBA) at a dose of 0.2% causes intestinal damage in piglets (Pan et al., 2013, 2018). In poultry and fish, the ingestion of raw soybean resulted in lower weight gains due to indigestion, a result of the morphological changes caused by the action of lectin in the intestine (Babot et al., 2021).

Among the antinutritional factors, protease inhibitors stand out, which act by blocking digestive enzymes such as trypsin and chymotrypsin. These compounds reduce protein digestibility and cause a compensatory increase in pancreatic enzyme secretion, which can lead to hypertrophy and hyperplasia of the pancreas in monogastric animals (Liener, 1994). The main protease inhibitors present in soybean are the Kunitz-type trypsin inhibitor (KTI) and the Bowman-Birk-type trypsin inhibitor (BBI), which differ in structure, abundance, and thermal stability. The Kunitz-type trypsin inhibitors (KTI) are one of the main antinutritional factors, present in the proportion of 4–15 mg of trypsin inhibitor per gram of soybean flour (Park et al., 2023). KTI is a monomeric protein that weighs 21kDa, has two disulfide bonds and 181 amino acid residues. The trypsin inhibitor binds to the Asp189 region of trypsin through a positively charged amino acid, which can be a lysine or arginine, and prevents its activity (Sweet et al., 1974; De Meester et al., 1998; Song and Suh, 1998). Soybean harbors a multigene family encoding Kunitz-type trypsin inhibitors. The predominant seed-expressed inhibitor is encoded by *KTI3* (Glyma.08G341500), located on chromosome 8, whereas related paralogs such as *KTI1* (Glyma.01G095000) and *KTI2* (e.g., Glyma.08G341400 in the reference genome) are located in distinct genomic regions, indicating that KTI genes contributing to trypsin inhibitory activity are not organized as a single tandem cluster (Jofuku and Goldberg, 1989; Zhang et al., 2021; Tian et al., 2025). The effects of Kunitz-type trypsin inhibitors on the digestibility of amino acids, especially lysine, methionine, and threonine, and on weight gain in livestock are well-documented, being consistently associated with reduced zootechnical performance (Herkelman et al., 1993; Palacios et al., 2014; Kuenz et al., 2022). This reduction results from the inhibition of pancreatic proteases, which stimulates the excessive secretion of digestive enzymes and increases endogenous amino acid losses, compromising the efficiency of dietary protein utilization (Herkelman et al., 1993; Perez-Maldonado et al., 2003; Kuenz et al., 2022).

The Bowman-Birk-type trypsin inhibitor is another type of protease inhibitor; however, it is found in smaller quantities in soybean, approximately 2 to 4 mg.g^-1^ of dry soybean (Sessa and Wolf, 2001; Kim et al., 2024; Liu et al., 2025a). The Bowman-Birk trypsin inhibitor is a monomeric protein with a weight of 7–8 kDa that has two inhibitory loop sites that inhibit chymotrypsin (Phe, Leu, and Tyr) and trypsin (Lys or Arg) at independent sites; these inhibitory sites are stabilized by 7 to 8 cysteines (Gitlin-Domagalska et al., 2020). It has 7 disulfide bonds and 71 amino acid residues. There are at least 11 Bowman-Birk trypsin inhibitors in soybean; however, only three of them (Glyma.16G208900, Glyma.14G117700, and Glyma.09G158700) are more highly expressed in seeds (Kim et al., 2024). Recently, it was shown that this inhibitor, despite being in smaller quantity in the seed, is responsible for about 80% of chymotrypsin inhibition and 45% of total trypsin inhibition (Liu, 2024; Liu et al., 2025a). Furthermore, this protease inhibitor is not completely eliminated by heat treatment because its 7 disulfide bonds confer stability, maintaining the protein’s conformation upon heating (Sessa and Wolf, 2001; Qi et al., 2005; Sabotič et al., 2011). Similar to KTI, BBI inhibition also causes a reduction in animal performance and, as observed in the literature, its magnitude is even greater than that observed for the KTI inhibitor (Moreau et al., 2024; Kim et al., 2025).

Phytic acid (myo-inositol hexafosfato, IP_6_) is the main storage form of phosphorus in soybean and other legume seeds, being essential for the plant’s initial development. However, this compound has a significant antinutritional effect because it forms insoluble complexes with minerals such as zinc (Zn), iron (Fe), and calcium (Ca), reducing their bioavailability for humans and animals (Graf and Eaton, 1984; Zhou et al., 1992; Punjabi et al., 2018). Phytic acid biosynthesis occurs in a pathway composed of four enzymatic reactions. First, glucose-6-phosphate is converted to myo-inositol-3-phosphate by the enzyme Myo-inositol-3-phosphate synthase (*MIPS1* – Glyma.10G123400) (Redekar et al., 2017; DeMers et al., 2021). Next, Myo-inositol kinase (MIK – Glyma.05G098300) phosphorylates myo-inositol, forming inositol monophosphate (Hegeman et al., 2001; Jin et al., 2021). The subsequent phosphorylations, performed by inositol polyphosphate kinases (IPK1 – Glyma.12G149100; IPK2 – Glyma.12G149200), result in the production of IP_6_ (Jin et al., 2021). Finally, Multidrug resistance-associated protein (MRP – Glyma.13G172300) transporters store the phytate in the seed vacuoles, ensuring phosphorus reserve and compound stability during development (Sashidhar et al., 2020). In addition to reducing mineral availability, phytate can also interact with proteins and digestive enzymes, such as trypsin and α-amylase, compromising the digestibility of proteins and starch (Bye et al., 2013; Theodoropoulos et al., 2018).

The raffinose family oligosaccharides (RFOs), such as raffinose and stachyose, are synthesized in soybean seeds by enzymes like galactinol synthase (GOLS), raffinose synthase (RS), and stachyose synthase (STS), whose genes are highly expressed during seed maturation (Dierking and Bilyeu, 2008; Valentine et al., 2017; de Koning et al., 2021; Jha et al., 2022). During germination and imbibition, the raffinose family oligosaccharides (RFOs) are degraded by α-galactosidases (α-GAL), which hydrolyze their α-1,6-galactosidic bonds, converting them into simple sugars like sucrose and galactose, providing energy to the developing embryo (de Koning et al., 2021). The total content of RFOs in soybean can exceed 40 mg.g^-1^with stachyose being the most predominant sugar (Elango et al., 2022). When consumed by monogastric animals, these oligosaccharides are not digested due to the absence of endogenous α-galactosidases, passing intact through the intestinal tract to the large intestine, where they are fermented by bacteria, resulting in gas production, intestinal discomfort, and reduced weight gain (Dierking and Bilyeu, 2008; Zeng et al., 2021; Elango et al., 2022).

Saponins are triterpenoid or steroid glycosides widely distributed in plants. In soybean, they mainly derive from soyasapogenols A, B, and E, formed from the β-amyrin precursor through the sequential action of oxygenases (CYPs) and glycosyltransferases (UGTs) (Shibuya et al., 2006; Sayama et al., 2012; Yano et al., 2018; Sundaramoorthy et al., 2019). These compounds conjugate with sugars to generate different soyasaponins, which accumulate mainly in the seed coat and hypocotyl of the seeds. In plants, they exhibit antifungal and antibacterial activity, in addition to reducing herbivory due to low palatability (Timilsena et al., 2023). However, they are considered antinutritional factors because they form foam, impart a bitter taste, and can interact with membranes and minerals, reducing feed intake and weight gain in monogastric animals (Timilsena et al., 2023).

Antinutritional factors can cause alterations in metabolism when consumed without the proper treatment (Liener, 1994); thermal treatment is conventionally used for the elimination of ANFs in animal feed. However, this treatment increases the energy cost of grain processing and can, as a result of using high temperatures, decrease the nutritional value and solubility of the grains (Bonturi et al., 2022). Several modifications for limiting antinutritional factors are summarized in **Table 1**.

**Table 1:** Reduction of antinutritional factors in soybean using biotechnology tools.

Biotechnology tool	Antinutritional factor	Gene	Results	Reference
CRISPR/Cas9	Protease inhibitors (BBI)	*BBI*	Drastic reduction in trypsin and chymotrypsin inhibitory activity.	Kim et al., 2024
	Protease inhibitors (KTI)	*KTI1*, *KTI3*	Significant decrease in trypsin inhibition.	Wang et al., 2023
	Phytate	*IPK1*	Reduced phytic acid; improved Fe and Zn bioavailability.	Song et al., 2022
	Raffinose family oligosaccharides (RFOs)	*GOLS*	35% reduction in RFOs; 41% increase in raffinose.	Le et al., 2020
	Raffinose family oligosaccharides (RFOs)	*RS2*, *RS3*, *RS4*	Nearly complete elimination of raffinose and stachyose.	Cao et al., 2022; Lin et al., 2023
	Saponins	*BAS1*, *BAS2*	Total absence of saponins in seeds and roots.	Asa et al., 2025
	Lectins	*Le1*	Truncated seed lectin protein with no biological activity	Kafer et al., 2026
	Lectins, KTI, Gly Bd 30k	*Le1, KTI3, P34*	Multiplex editing of 3 ANFs, generating an hypoallergenic phenotype	Liu et al., 2025b
RNAi	Protease inhibitors	*BBI*, *KTI*	2.8–6× lower trypsin inhibition; up to 80% lower chymotrypsin inhibition.	Kim et al., 2025
	RFOs	*RS2*	Reduction of 82.5% in raffinose and 68.1% in stachyose.	Valentine et al., 2017
	Phytate	*MIPS1*	41.3% reduction in seed phytate; normal plant development.	Nunes et al., 2006; Kumar et al., 2019
	Phytate	*IPK2*	Lower phytate, higher mineral availability.	Punjabi et al., 2018
Mutagenesis (EMS)	Phytate and RFOs	—	Mutants with low-phytate and reduced oligosaccharides.	Hitz et al., 2002
	Phytate	—	Stable low-phytate soybean lines.	Yuan et al., 2007
	Lectin	—	Identification of low-lectin mutants.	George et al., 2008
	Saponins	—	Modified saponin biosynthesis via UGT alterations.	Sundaramoorthy et al., 2019

## 4 Protein

Soybean has always been traded quantitatively and not based on its quality. Therefore, historically, genetic improvement has always prioritized increasing plant productivity at the expense of grain quality (Lee et al., 2019), as there is a negative correlation between protein content and productivity (Lee et al., 2019; Zhang et al., 2021). Furthermore, the protein content is also negatively related to the oil content of the seed, which further hinders the manipulation of this attribute by genetic improvement (Hymowitz, 1970). This becomes more serious with the decrease in protein content over the years due to genetic improvement efforts for increased productivity. More recent soybean cultivars show a protein content of around 36%, while the ideal level considered for the industry is 42% (Medic et al., 2014; Patil et al., 2017; Assefa et al., 2018).

Soybean grain quality for animal feed is determined by the coordinated regulation of a limited number of core metabolic pathways, including carbon allocation, nitrogen assimilation, phosphorus storage, and the biosynthesis of antinutritional factors. Carbon allocation during seed filling controls the partitioning of sucrose between oil and protein biosynthesis, thereby directly influencing the oil–protein balance (Li et al., 2024). Nitrogen assimilation and transport regulate amino acid availability for storage protein synthesis, defining both protein content and quality (Kumar et al., 2025; Shi et al., 2025). Phosphorus storage pathways, mainly associated with phytic acid accumulation, affect mineral bioavailability and protein digestibility in animal diets. In parallel, secondary metabolic pathways lead to the accumulation of antinutritional compounds such as protease inhibitors, raffinose family oligosaccharides, and saponins, which negatively impact nutrient utilization, particularly in monogastric animals (Yao et al., 2023).

Soybean proteins have been classified by ultracentrifugation, which identified four protein fractions: 2S, 7S, 11S, and 15S, according to the Svedberg index (Jegadeesan and Yu, 2020). The 2S fraction, which represents 8% to 22% of soluble soybean protein, contains various enzymes, including trypsin inhibitors, such as the Bowman-Birk and Kunitz inhibitors. Other proteins, such as lectins and lipoxygenases, represent 1% to 3% of the total soybean protein (Vasconcelos et al., 2006). The 15S fraction, although poorly characterized, corresponds to about 5% of the total soybean protein and is known to contain polymers of other soybean proteins. Most of the soybean storage proteins, β-conglycinin and glycinin, are present in the 7S and 11S fractions, respectively, representing about 35% and 54% of the soluble soybean protein (Capriotti et al., 2014; Din et al., 2021; John et al., 2021). Glycinin is composed of six subunits that form a hexameric structure of 360 kDa. The six subunits are composed of basic units (B1a, B1b, B2, B3, and B4) and acidic units (A1a, A1b, A2, A3, A4, and A5) that are linked by disulfide bonds. This protein is encoded by several genes that have been separated into groups: group 1 composed of genes Gy1 (*A1aB2*), Gy2 (*A2B1a*); group 2 composed of Gy4 (*A5A4B3*) and Gy5 (*A3B4*); and group 3 composed of two pseudogenes (gy6 and gy8); and Gy7 (group of unclassified polypeptides). Glycinin has a higher concentration of sulfur-containing amino acids, such as methionine and cysteine, in which soybean is relatively deficient (Patil et al., 2017). The 7S fraction is composed of β-conglycinin, which is a trimer of 180 kDa, with about 15 genes (*CG1–CG15*) encoding the protein. Several strategies to increase soybean protein and protein quality are presented in **Table 2**.

**Table 2:** Enhancement of protein content and nutritional quality in soybean through genetic engineering and molecular breeding.

Biotechnology tool	Target (Trait)	Gene	Results	Reference
CRISPR/Cas9	Protein–oil balance	*GmSWEET10a*, *GmSWEET10b*	Knockout increased protein by up to 32%, reduced oil by 40%; AlphaFold2 modeling confirmed structure–function link.	Wang et al., 2020; Wang et al., 2025a
	Protein accumulation	*GmEOD1*	Deletion increased seed protein from 37.9%- 39.8% without affecting sugars.	Yu et al., 2023
	Seed filling regulation	*GmMFT*	Knockout increased protein and reduced oil; overexpression had opposite effect.	Cai et al., 2023
	Protein accumulation	*NF-YC4*	Knockout of repressor sites in the promotor of NF-YC4 increased protein content in soybean by 11%	Wang et al., 2025b
	Protein accumulation	*RIC1a/RIC2a*	Knockout of these genes lead to increase nodulation and resulted in 1.77-4.42% increase in protein content.	Zhong et al., 2024
RNAi	Storage protein rebalancing	*β-conglycinin*	Silencing decreased β-conglycinin, with compensatory glycinin increase.	Kinney et al., 2001
	Storage protein rebalancing	*β-conglycinin*, *glycinin*	Double silencing caused global rebalancing of storage proteins with no protein loss.	Schmidt et al., 2011
	Carbon metabolism (invertase regulation)	*CIF1*	Increased invertase activity; higher soluble sugars, starch, and protein in mature seeds.	Tang et al., 2016
RNAi/CRISPR	Seed development regulation	*AIP2a*, *AIP2b*	Up to +7 percentage points increase in seed protein content.	Shen et al., 2022
Mutagenesis/Breeding	Amino acid biosynthesis	*OASS* (O-acetylserine sulfhydrylase)	Lines with 41–47% total protein; higher cysteine and methionine levels.	Alaswad et al., 2021
Transgenesis	Sulfur amino acid biosynthesis	*GmCGS2* (cystathionine gamma-synthase 2)	Overexpression led to significant increases in methionine and total amino acid content in mature seeds.	Zhang et al., 2023
	Sulfur amino acid enrichment	*proglycinin* (A1aB1b), *MB-16*	16–65% increase in sulfur-containing amino acids (Met + Cys).	El-Shemy et al., 2007; Zhang et al., 2014

Advances in molecular genetics and metabolic engineering have demonstrated that targeted manipulation of these pathways can shift metabolic fluxes toward higher protein content and lower antinutritional factor accumulation, without necessarily compromising seed viability (Yao et al., 2023). Consequently, genome editing and related biotechnological tools have emerged as effective approaches to directly modulate key enzymatic steps, transport processes, and regulatory nodes controlling soybean grain composition.

## 5 Biotechnological strategies to improve the quality of soybean protein for animal feed

### 5.1 Antinutritional factors

#### 5.1.1 Classic plant breeding

Various genetic mapping studies have identified QTLs and mutations associated with the reduction or elimination of antinutritional factors in soybean, which has been effectively exploited in breeding programs. In particular, Choi et al. documented the accumulation and fixation of recessive alleles for antinutritional components, leading to the creation of tetra-null and penta-null lines that combine the absence/low activity of *KTI* and lectin with lipoxygenase deletions and reduction of other antinutrients, demonstrating the applicability of marker-assisted selection in conventional crossing schemes (Choi et al., 2021, 2022). Regarding the raffinose family oligosaccharides (RFOs), QTLs on chromosomes 10 and 11 control the raffinose and stachyose contents, and candidate genes such as Glyma.10G154400 and Glyma.11G136200 are directly associated with the phenotypic variation (He et al., 2025).Finally, stable QTLs related to saponin content were mapped on chromosomes 5 and 6 near the SSR markers Satt384 and Sat_312, controlling the total content and the aglycone profile (Sayama et al., 2012; Teraishi et al., 2017). Despite advances in genetic breeding, there is a challenge in accumulating different desirable characteristics without pleiotropic effects, such as reduced yield and altered growth habit. Given this context, other strategies have been employed to reduce the presence of antinutritional factors in soybeans.

#### 5.1.2 Mutagenesis

The generation of genetic variability through mutagenic agents has been widely used as an alternative to obtain mutants with specific characteristics and increase the genetic diversity available for soybean breeding (Li et al., 2017). Compounds like ethyl methanesulfonate (EMS) have been employed in several studies to induce large-scale point mutations, allowing the identification of variants useful for the reduction of antinutritional factors, such as phytic acid, lectins, saponins, raffinose family oligosaccharides (RFOs), and protease inhibitors (Hitz et al., 2002; Yuan et al., 2007; George et al., 2008; Sundaramoorthy et al., 2019). Although extremely valuable, these mutant populations present significant limitations: the high frequency of random mutations and the lack of specific targeting make the screening process laborious and time-consuming, requiring extensive phenotypic analyses and subsequent genotypic validation to confirm the target gene. Furthermore, the accumulation of secondary mutations can introduce undesirable pleiotropic effects, making the direct association between genotype and phenotype difficult (Hitz et al., 2002; Yuan et al., 2007; George et al., 2008; Li et al., 2017; Sundaramoorthy et al., 2019). In contrast, modern genetic engineering tools, such as genome editing based on programmable nuclease systems (CRISPR, TALEN, RNAi), allow the generation of precise and heritable mutations in specific genes, eliminating the need for massive screenings and accelerating the process of functional validation.

#### 5.1.3 RNAi

The use of RNA interference (RNAi) has been a powerful tool for reducing the expression of genes that produce antinutritional factors. RNAi reduces gene expression through the selective degradation of the target messenger RNA (mRNA). This occurs when small double-stranded RNAs (siRNAs) are generated from a sequence complementary to the gene of interest, leading the RNA-induced silencing complex (RISC) to recognize and cleave the corresponding mRNA, preventing its translation into protein. With respect to protease inhibitors, interesting results have been obtained by decreasing the expression of the KTI and BBi genes, resulting in a 2.8-6x reduction in inhibitory activity for trypsin, and a reduction of up to 80% for chymotrypsin (Kim et al., 2025). For RFOs, by targeting the RS2 gene, transgenic lines showed a reduction of 82.5% and 68.1% for raffinose and stachyose, respectively (Valentine et al., 2017).Regarding phytic acid, significant reductions were obtained when the *MIPS1* (myo-inositol-1-phosphate synthase) gene was silenced by RNAi. However, initial studies reported severe pleiotropic effects, including seed development failures, despite the drastic decrease in phytate content observed in the transgenic lines (Nunes et al., 2006). Subsequently, Kumar et al., 2019 demonstrated that specific silencing for seed tissue allows for reductions of up to 41.34% in phytate content, without interfering with plant growth and development. Similarly, the suppression of the *IPK2* gene by RNAi also resulted in lower phytate accumulation and higher mineral availability in the seeds, without compromising plant development or viability (Punjabi et al., 2018).

#### 5.1.4 CRISPR

Although genome editing represents a common technological platform, its application differs substantially depending on the biological nature of the target trait. In the case of antinutritional factors, which are undesirable components of the final feed product, genome editing strategies are predominantly based on direct gene knockouts aimed at preventing protein or metabolite accumulation in the seed. Because these compounds are dispensable for seed nutritional performance, loss-of-function mutations often result in clear phenotypic gains with limited pleiotropic effects.

In this context, several studies have demonstrated the effectiveness of knockout-based strategies to eliminate major antinutritional factors in soybean seeds. Early CRISPR-based approaches primarily targeted individual antinutritional proteins. For protease inhibitors, Kim et al. (2024) developed mutant plants with reduced trypsin and chymotrypsin activities through loss-of-function mutations in Bowman–Birk inhibitor (*BBI*) genes. Similarly, Wang et al. (2023) generated *KTI1* and *KTI3* knockout lines, resulting in a significant decrease in Kunitz trypsin inhibitor activity.

Parallel efforts focused on reducing antinutritional carbohydrates. Knockout of raffinose synthase and stachyose synthase genes (*RS2, RS3*, and *RS4*) led to an almost complete elimination of raffinose family oligosaccharides (RFOs) from soybean seeds (Cao et al., 2022; Lin et al., 2023). Consistently, disruption of the GOLS gene, encoding galactinol synthase, reduced total RFO content by 35.2% and stachyose by 35.4%, with a compensatory increase in raffinose levels (Le et al., 2020).

Seed lectins have also emerged as clear targets for knockout-based genome editing. CRISPR-mediated mutagenesis of the *Le1* gene generated elite soybean lines expressing a truncated, non-functional lectin, resulting in the complete elimination of lectin activity in seeds (Kafer et al., 2026). Building on these single-target approaches, Liu et al. (2025b) employed CRISPR/Cas12a-mediated multiplex genome editing to simultaneously disrupt Kunitz trypsin inhibitor (*KTI*), soybean agglutinin (*Le1*), and the major allergenic protein P34 (Liu et al., 2025b). The resulting soybean lines exhibited markedly reduced protease inhibitory activity and allergenicity, demonstrating that multiplex editing can efficiently combine multiple desirable traits related to feed and food quality within a single genetic background.

In addition to proteins and carbohydrates, genome editing has also been applied to other antinutritional pathways. Song et al., 2022 reported that knockout of the *IPK1* gene, encoding myo-inositol 1,3,4,5,6-pentakisphosphate kinase, significantly reduced phytic acid accumulation in seeds, thereby increasing the bioavailability of micronutrients such as iron and zinc. Most recently, targeted mutagenesis of *BAS1* and *BAS2*, genes involved in β-amyrin biosynthesis, resulted in the complete absence of saponins in seeds, leaves, and young roots, as confirmed by HPLC analysis (Asa et al., 2025). While antinutritional factors can often be efficiently eliminated through direct gene knockouts, improving seed protein content represents a fundamentally different genome editing challenge, requiring strategies that modulate regulatory nodes and metabolic fluxes rather than abolishing specific gene functions.

### 5.2 Protein

#### 5.2.1 Classic plant breeding for protein

Despite the importance of soybean grain protein, increasing its content is often overlooked in genetic breeding programs in favor of increasing yield, which is the main objective (Patil et al., 2017; Singer et al., 2019; Gupta and Manjaya, 2022) This occurs due to the inverse relationship between protein content and other traits, such as oil content and yield (Warrington et al., 2015). Furthermore, soybean protein content is controlled by multiple genes, which makes its manipulation difficult. Consequently, more recent soybean cultivars have shown reduced protein content compared to their progenitors.

The main genetic breeding tools for identifying genomic regions of interest use Genome-Wide Association Study (GWAS) and Linkage Analysis. These approaches allow mapping genomic regions and identifying Quantitative Trait Loci (QTLs) associated with traits of interest, which are subsequently used in genetic breeding, especially in Marker-Assisted Selection (MAS). Currently, there are about 249 QTLs associated with protein content, and 16 have been approved by the Soybean Genetics Committee (Grant et al., 2010; Brown et al., 2021; Soybase).One of the first QTLs for oil and protein was identified by linkage analysis, mapping the region of chromosomes 20 and 15 using RFLP markers, with the Glycine max line A81-356022 (high oil content and low protein content) and the Glycine soja PI (high protein content and low oil content) (Diers et al., 1992)Subsequently, several studies expanded the knowledge about these QTLs, which are considered the best characterized in relation to oil and protein (Nichols et al., 2006; Bolon et al., 2010; Singer et al., 2019; Gupta and Manjaya, 2022). Warrington et al. (2015) found that the allele of PI 619083 (high protein content) on chromosome 20 is responsible for 55% of the phenotypic variation in the population obtained from the cross of Benning times Danbaekkong.

The genomic region of the QTL was narrowed to a window smaller than 1 Mb based on GWAS (Vaughn et al., 2014). Subsequently, Bandillo et al., 2015 restricted the chromosome 20 region to only three possible targets in the soybean reference genome. Among these candidates, the gene Glyma.20G085100 encodes a CCT domain (CONSTANS, CO-LIKE, and TOC1), which is related to photoperiod regulation and flowering (Liu et al., 2020). It was observed that in Glycine soja PIs and their progenies, there is a 321 bp deletion in the third exon, while in Glycine max, a transposable element insertion occurred (Liu et al., 2020; Fliege et al., 2022; Goettel et al., 2022; Marsh et al., 2022).The QTL on chromosome 15 was mapped to the gene Glyma.15G049200, which encodes the sugar transporter SWEET39 (Sugars Will Eventually be Exported Transporters), primarily expressed in the seed coat and parenchyma, regulating oil and protein accumulation by delivering sugars from the maternal tissue to the embryo. It was found that the allele favorable to higher protein content has a CC insertion, while the allele favorable to oil content has a deletion of these two nucleotides, altering the protein reading frame and resulting in a truncated protein (Zhang et al., 2020). Other transporters, such as AVT3 (Amino Acid Vacuolar Transporter), CAT9 (Cationic Amino Acid Transporters), UMAMIT25 (Usually Multiple Acids Move In And Out Transporters 25), and UPS2 (Ureide Permease 2), were also associated with higher protein content (Besnard et al., 2018; Grant et al., 2021; Joaquim et al., 2022).

#### 5.2.2 Classic plant breeding for protein quality

Another important point for soybean breeding is amino acid quality. Soybean naturally has a reduced content of sulfur-containing amino acids, such as methionine and cysteine, which need to be supplemented in the diet when soybean is used as animal feed. The content of sulfur-containing amino acids is a complex trait, governed by multiple genes and affected by the environment, which makes its manipulation difficult through genetic breeding. Several studies have identified possible targets to increase the content of these amino acids through GWAS and Linkage Analysis (Panthee et al., 2006; Ma et al., 2019; Yuan et al., 2021; Zhong et al., 2023) About 126 QTLs have been registered that associate the content of methionine and cysteine in soybean (Grant et al., 2010; Soybase).

Yuan et al. (2021) conducted a GWAS for the amino acids cysteine, methionine, and cysteine + methionine in a population of 165 soybean accessions, using a high-density SNP array. 138 SNPs associated with these amino acids and two candidate genes on chromosome 7, related to the glycine protein family, which may affect the metabolism of sulfur-containing amino acids, were identified. The expression of these genes was observed only in seeds, not occurring in other tissues, through RT-qPCR.

Similarly, Zhong et al. (2023) used linkage analysis derived from the genotypes Guizao 1 (high sulfur-containing amino acid content) and Brazil 13 (low sulfur-containing amino acid content) to evaluate QTLs for sulfur-containing amino acids. Among the 44 QTLs observed, those found on chromosome 5 were stable across multiple environments, being linked to the 11S, 7S fractions and the sulfur-containing amino acid content. To validate the genes, the authors performed RNA-Seq in the parental seeds at three different stages of development, observing genes responsible for the biosynthesis of methionine, thioredoxin, and albumin, which showed higher expression in Brazil 13. This higher expression was related to genes linked to yield and 100-grain weight, which have a negative correlation with protein.

Alaswad et al., 2021 made use of introgression, using a line that overexpresses the enzyme O-acetylserine sulfhydrylase (CS) in a Korean cultivar with high protein content (Lee). The authors observed that the recombinant lines showed protein content varying between 41.3% and 47.7%, while the parental lines varied from 34.8% (CS) to 44.7% (Lee). Regarding sulfur-containing amino acids, a higher content was observed in the mutant lines, varying from 1.1% to 1.26%, while the parental lines showed 0.79% (Lee) and 1.1% (CS). Furthermore, SDS-PAGE and Western blot analyses revealed the accumulation of Bowman-Birk proteinase inhibitors and lunasin, protein fractions rich in sulfur-containing amino acids.

#### 5.2.3 Transgeny

Transgenesis has been widely employed in soybean with the goal of improving protein quality, especially by increasing the levels of methionine and cysteine, essential amino acids naturally limiting in the crop. El-Shemy et al., 2007 introduced a synthetic proglycinin (A1aB1b) gene containing four consecutive methionine residues into soybean, which resulted in greater accumulation of glycinin in the transgenic seeds. Similarly, the introduction of the synthetic MB-16 protein, enriched in methionine, threonine, lysine, and leucine, promoted a 16.2% increase in methionine and a 65.9% increase in cysteine in mature seeds, even with the absence of the recombinant protein in the final stage of development (Zhang et al., 2014). Another strategy used to increase the content of these amino acids consists of the overexpression of genes involved in the biosynthesis of methionine and cysteine. The overexpression of the O-acetylserine sulfhydrylase (*OASS*) gene increased expression during seed development, resulting in an elevation of 58–74% in protein-bound cysteine and 22–32% in the free form (Kim et al., 2012). Similarly, the overexpression of the cystathionine gamma-synthase 2(*GmCGS2*) gene also promoted a significant increase in the levels of methionine and other amino acids in mature seeds (Zhang et al., 2023).

#### 5.2.4 RNAi

Gene silencing by RNAi has been widely employed to modify the protein profile of soybean seeds, allowing the phenomenon of storage protein rebalancing to be observed (Herman, 2014). One of the first knockdown studies was conducted on the β-conglycinin storage protein, resulting in its lower expression; however, the total protein content of the seed remained unchanged, due to the compensatory increase of the other main storage protein, glycinin (Kinney et al., 2001). Subsequently, the simultaneous silencing of β-conglycinin and glycinin promoted a global rebalancing of storage proteins, generating seeds with a significantly altered protein profile, although without expressive changes in the total protein content (Schmidt et al., 2011).

On the other hand, the strategy of silencing negative regulators of metabolism and seed development has proven effective in increasing the total protein content. A notable example is the silencing of the *CIF1* (cell wall invertase inhibitor 1) gene, which encodes an inhibitor of cell-wall invertase, an enzyme responsible for the conversion of sucrose into glucose and fructose, sugars essential for the signaling and synthesis of biomolecules during seed filling. The RNAi-CIF1 plants exhibited higher invertase activity, resulting in superior accumulation of soluble sugars, starch, and protein in mature seeds (Tang et al., 2016). Similarly, the simultaneous silencing of the two copies of the *E3-RING ubiquitin ligase AIP2* gene (*AIP2a and AIP2b*), a negative regulator of the *ABI3* transcription factor, essential for proper seed filling, led to an increase of up to seven percentage points in the seed protein content (Shen et al., 2022).

#### 5.2.5 CRISPR

In contrast, improving seed protein content represents a more nuanced metabolic challenge. Protein accumulation is a complex quantitative trait tightly connected to carbon and nitrogen fluxes, seed development, and source–sink relationships. In this context, genome editing strategies are more effective when targeting negative regulatory genes or modifying the activity of transporters and key metabolic nodes, rather than eliminating structural genes. Such interventions enable a redistribution of metabolic resources toward protein synthesis while preserving seed viability and agronomic performance.

A clear example of this strategy is provided by studies on sugar transporters of the SWEET family. Wang et al., 2020 identified GmSWEET10α and GmSWEET10β as central regulators of carbon allocation during soybean seed development. CRISPR/Cas9-mediated knockout of GmSWEET10α and GmSWEET10β resulted in smaller grain size, with reductions of 7.4% and 7.2% in 100-seed weight and fatty acid content, respectively, accompanied by an increase of 6.4% in seed protein content compared to the non-edited cultivar. In contrast, overexpression lines exhibited larger grains with significantly increased oil content and reduced protein levels, reinforcing the trade-off between carbon partitioning toward lipid or protein biosynthesis. Notably, the simultaneous knockout of GmSWEET10α and GmSWEET10β in different genetic backgrounds further amplified this effect, leading to an increase of up to 32.1% in protein content, together with reductions of 40.7% in fatty acid content and 40.2% in 100-seed weight, highlighting the pivotal role of sucrose transport in controlling the oil–protein balance during seed filling. Similar metabolic trade-offs have been reported for the MOTHER-OF-FT-AND-TFL1 (*GmMFT*) gene, further supporting the role of regulatory factors in coordinating seed size, oil deposition, and protein accumulation.

Further evidence supporting the role of regulatory genes in controlling soybean seed protein content is provided by studies on MOTHER-OF-FT-AND-TFL1 (GmMFT). When compared with the wild type, two GmMFT-silenced lines of the Young variety displayed a significant increase in grain protein content, whereas overexpression of the same gene resulted in a reduction in the proportion of stored protein. These genetic manipulations were also associated with changes in seed size and morphology. While the wild-type plants exhibited an average 100-seed weight of 13.8 g, overexpression lines showed increased seed weights of 16.9 g and 16.1 g, whereas knockout lines exhibited reduced weights of 11.3 g and 10.6 g.

Consistent with these phenotypic observations, transcriptomic analyses across diverse soybean germplasms revealed a strong association between GmMFT expression levels and grain oil content. Higher GmMFT expression was consistently observed in nine genotypes with oil contents ranging from 21.13% to 22.35%, whereas significantly lower expression levels were detected in eight genotypes with oil contents between 18.02% and 19.59%. Together, these data indicate that differential GmMFT expression plays a central role in coordinating oil and protein accumulation as well as grain weight during seed development (Cai et al., 2023).

Recent advances in cis-regulatory genome editing have further expanded strategies for protein improvement. CRISPR/Cas9-based editing of the NF-YC4 promoter in soybean was used to remove a cis-regulatory element associated with transcriptional repression, resulting in increased NF-YC4 expression during seed development. This targeted derepression led to a significant increase in seed protein content while preserving agronomic performance, demonstrating that precise modification of regulatory elements can redirect metabolic fluxes toward protein accumulation without disrupting structural genes (Wang et al., 2025b).

A similar regulatory effect was observed for *GmEOD1*, which encodes an E3 ubiquitin ligase. CRISPR/Cas9-mediated deletion of *GmEOD1* in the Jack soybean cultivar resulted in grains with a protein content of 39.84%, significantly higher than that of the control (37.96%; p < 0.01). This increase in protein content was accompanied by a modest but significant reduction in oil content (20.82% in the mutant versus 21.33% in the control; p < 0.05), while soluble reducing sugar levels remained unchanged. Notably, the combined content of crude protein and oil increased from 59.28% in the control to 60.66% in the edited lines (p < 0.05). In addition, daidzin levels were significantly reduced in the mutant seeds (p < 0.05), whereas the contents of palmitic, stearic, oleic, linoleic, and linolenic acids, as well as total saturated and unsaturated fatty acids, were not significantly altered (Yu et al., 2023).

While most genome editing efforts to enhance soybean protein content have focused on seed-specific regulators, recent evidence indicates that manipulating whole-plant physiological processes can also influence grain composition. Beyond targeting storage protein regulators and transporters, genome editing can impact seed composition through modulation of whole-plant physiology. For instance, moderate enhancement of nodulation via editing of *RIC1a/RIC2a* genes in soybean increased both grain yield and protein content, demonstrating that editing genes involved in carbon–nitrogen balance can indirectly improve seed nutritional traits under field conditions (Zhong et al., 2024).

An innovative approach combined the use of CRISPR/Cas9 and structural modeling by AlphaFold2 to generate different soybean lines with variations in protein and oil content, through modifications in the GmSWEET10α and GmSWEET10β genes. The study demonstrated that structural alterations in the C-terminal region of these proteins modify the conformation of the sucrose transport tunnel, controlling its efficiency. When the tunnel remains more open, the capacity for sugar transport from the seed coat to the embryo increases, resulting in greater oil accumulation and lower protein content in the seeds. In contrast, mutations that reduce the opening of the transport pocket decrease the sugar flow, leading to reduced oil content and a compensatory increase in protein. This approach, guided by AlphaFold2, illustrates how artificial intelligence tools can guide targeted and predictable gene edits, allowing the creation of new structural variants of proteins with agronomic potential and expanding the genetic variability available for soybean breeding (Wang et al., 2025a).

## 6 Conclusion and future perspectives

**Figure 2:**
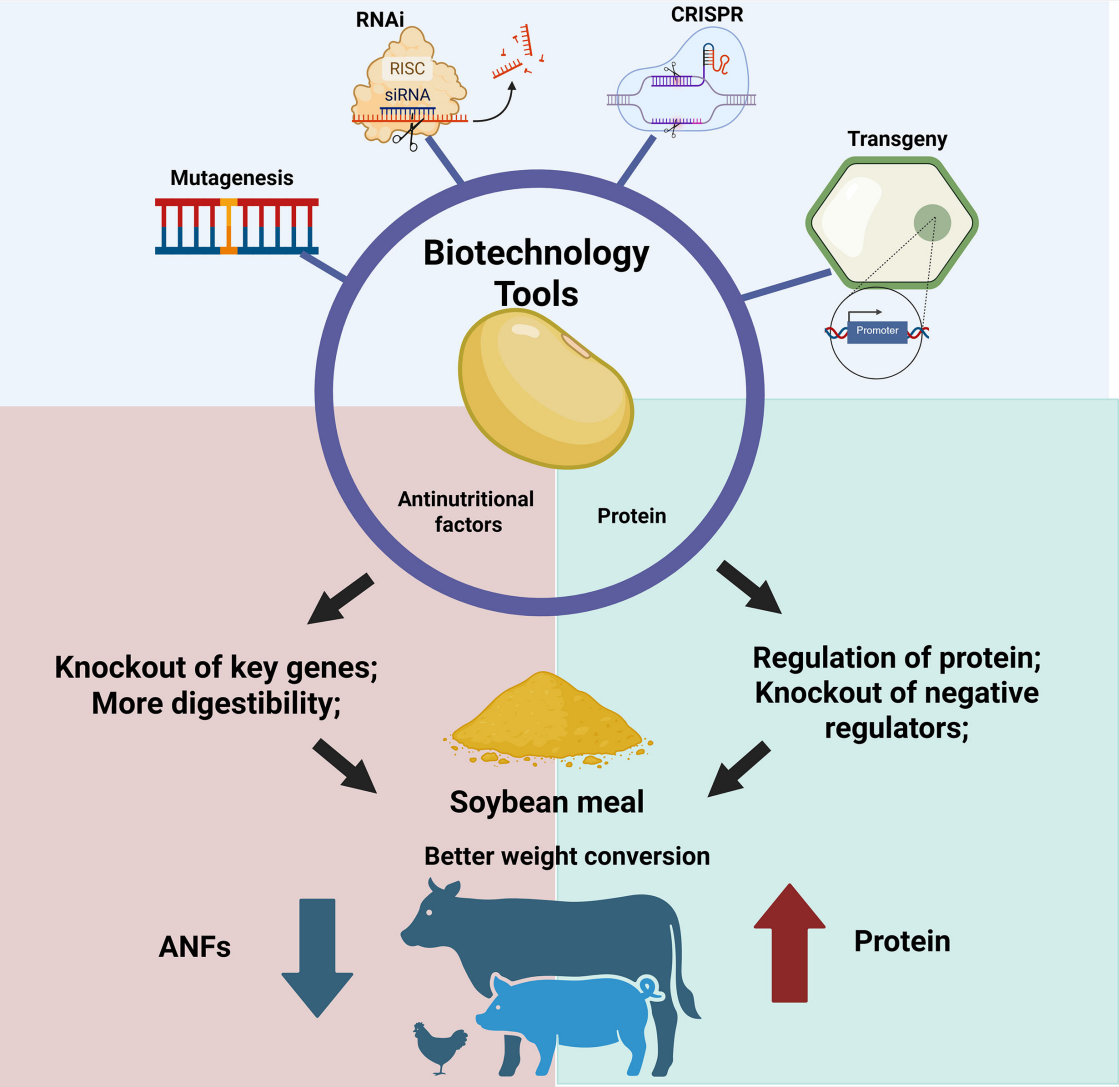
Biotechnology tools for reducing antinutritional factors and enhancing protein content in soybean.

The advancement of biotechnology tools and computational biology has considerably expanded the possibilities for soybean improvement aimed at increasing protein content and enhancing the nutritional quality of the seeds. Emerging technologies, such as CRISPR/Cas guided by protein structural modeling, rational promoter engineering, and Prime Editing, represent a new frontier in the precise manipulation of genes related to seed storage metabolism. The application of the biotech tools to enhance digestibility and protein levels in soybean meal are presented in **Figure 2**.

A recent example is the work by Wang et al. (2025a), which combined the CRISPR/Cas9 system with structural predictions obtained through AlphaFold2 to edit the genes *GmSWEET10a* and *GmSWEET10b*, responsible for sucrose transport between the seed coat and the embryo. Alterations in the C-terminal structure of these proteins modified the shape of the transport tunnel, resulting in lines with different sugar translocation capacities. Variants with a more open tunnel showed higher oil accumulation and lower protein content, while mutations that restricted sucrose passage increased protein content. This integration of structural prediction and gene editing paves the way for protein engineering guided by molecular modeling, capable of generating genetic variability under rational control.

Another promising strategy is promoter engineering, which allows spatial and temporal modulation of genes related to nitrogen metabolism, storage protein synthesis, and carbon transport. The use of strong, seed-development-specific promoters can direct metabolic resources toward protein synthesis without compromising yield. This approach has already been tested in other legumes and could be applied to optimize the expression of key genes in soybean, minimizing undesirable pleiotropic effects.

Finally, Prime Editing, an evolution of the CRISPR system, emerges as a high-potential tool for precision breeding. This technique combines the action of a reverse transcriptase guided by RNA with a modified Cas endonuclease, allowing precise point mutations, insertions, and deletions without generating double-strand DNA breaks. Applying this technology in soybean could enable the controlled introduction of favorable variants in genes related to protein accumulation, structural stability of storage proteins, and the efficiency of sugar and amino acid transport, overcoming limitations observed in previous approaches based on random mutagenesis or RNAi.

The future of soybean genetic improvement, therefore, moves toward an integration of high-precision genome editing, AI-assisted structural modeling, and refined gene regulation, enabling the development of cultivars with superior nutritional profiles, adapted to the demands of industry and productive sustainability.
